# Rheumatoid cachexia is associated with dyslipidemia and low levels of atheroprotective natural antibodies against phosphorylcholine but not with dietary fat in patients with rheumatoid arthritis: a cross-sectional study

**DOI:** 10.1186/ar2643

**Published:** 2009-03-10

**Authors:** Ann-Charlotte Elkan, Niclas Håkansson, Johan Frostegård, Tommy Cederholm, Ingiäld Hafström

**Affiliations:** 1 Karolinska Institute at the Department of Rheumatology, Karolinska University Hospital Huddinge, 141 86 Stockholm, Sweden; 2 The National Institute of Environmental Medicine, Division of Nutritional Epidemiology, Karolinska Institute, 171 77 Stockholm, Sweden; 3 Karolinska Institute at the Department of Medicine, Karolinska University Hospital Huddinge, 141 86 Stockholm, Sweden; 4 Department of Public Health and Caring Science/Clinical Nutrition and Metabolism, Uppsala University, 751 85 Uppsala, Sweden

## Abstract

**Introduction:**

Patients with rheumatoid arthritis (RA) have an increased risk for cardiovascular disease (CVD) independent of traditional risk factors. The aim of this study was to analyze the associations between diet, body composition, lipids and atheroprotective natural antibodies against phosphorylcholine (anti-PC) in patients with RA.

**Methods:**

A total of 80 RA patients (76% women), mean age (standard deviation (SD)) 61.4 (12) years and median disease duration of 6 years, were assessed by food frequency questionnaire (FFQ), fatty acid profile in adipose tissue and whole-body dual energy x ray absorptiometry (DXA). Rheumatoid cachexia was defined as fat free mass index below the 25th percentile and fat mass index above the 50th percentile of a reference population. Blood lipids, oxidized low-density lipoprotein (oxLDL) and anti-PC levels were determined.

**Results:**

The mean body mass index for the women and men was 25.0 and 27.0, respectively. Central obesity was found in 57% of the women (waist circumference >80 cm) and in 89% of the men (waist circumference >94 cm). In all, 18% of the women and 26% of the men had rheumatoid cachexia. These patients had significantly higher total cholesterol (*P *< 0.033), LDL (*P *< 0.029), and trendwise oxLDL (*P *= 0.056) as well as lower anti-PC IgM (*P *= 0.040), higher frequency of hypertension (69%) and metabolic syndrome (25%) than those without. The patients reported a high dietary intake of saturated fat, which partly correlated with fatty acid composition in adipose tissue and significantly with disease activity. However, patients with or without cachexia did not differ with respect to dietary fat intake or intake of Mediterranean-like diet. Additionally, patients on a Mediterranean-like diet had high levels of anti-PC (*P *< 0.001).

**Conclusions:**

About one in five patients with low-active RA displayed rheumatoid cachexia. This condition was associated with high levels of LDL cholesterol, low levels of atheroprotective anti-PC and high frequency of hypertension, which is of interest in the context of CVD in RA. The cachexia could not be related to diet fat intake. However, patients on a Mediterranean-like diet had high anti-PC levels in spite of similar frequency of cachexia. High anti-PC levels may provide some protection against CVD.

## Introduction

Rheumatoid arthritis (RA) is a chronic systemic inflammatory disease with higher mortality rates than observed in the general population [1,2]. This increased mortality is largely attributed to cardiovascular disease (CVD) [3]. The increase of CVD is suggested to be related to the effects of the chronic inflammation on the vascular endothelium, mainly through dysregulation of lipid metabolism.

Growing evidence points to inflammation in RA being associated with a worsening of the lipid profile [4,5], a factor already present early in the disease [6]. Dyslipidemia in RA is mainly presented by low concentrations of high-density lipoprotein (HDL), which is associated with an unfavorable cardiovascular risk. Total cholesterol and HDL levels in RA are inversely associated with the acute phase response, regardless of whether patients are treated with antirheumatic drugs or not. Furthermore, patients with RA have increased levels of oxidized low-density lipoprotein (oxLDL) in serum compared with healthy subjects, which may contribute to the increased risk of CVD in this patient group [7] as LDL oxidation probably has an important role in the pathogenesis of atherosclerosis [8].

Phosphorylcholine (PC) is a major ligand in oxLDL, exposed on platelet activating factor (PAF)-like phospholipids, which promote inflammation [9]. Antibodies against PC (anti-PC) of the IgM subclass are inversely associated with development of atherosclerosis in patients with established hypertension [10]. Further, low levels of anti-PC antibodies are associated with an increased risk of development of CVD [11].

In RA, anti-PC have not been studied in relation to CVD but we have recently shown that the level of anti-PC in serum increased when changing from a normal to a gluten-free vegan diet [12].

Another consequence of the course of RA disease is change in body composition, with reduced fat free mass (FFM), of which muscle mass is the largest component [13,14]. The decline in FFM is, in RA, often associated with increased fat mass (FM) and thus, with little or no weight loss, also with a maintained body mass index (BMI) [15,16]. This condition has been named 'rheumatoid cachexia' [13] and is believed to accelerate morbidity and mortality in RA [17].

Rheumatoid cachexia has been described in up to two thirds of RA patients and is suggested to be caused by cytokine-driven hypermetabolism and protein degradation [14,18]. However, it has also been found in patients with good disease control [14]. Another proposed cause is poor nutrition [19]. Dietary intake appears to be adequate in terms of energy and protein among patients with RA [18,20]. However, inadequate nutrient intake has also been reported [21,22]. Further support for a role of diet in the context of rheumatoid cachexia can be found in a recent report that addition of high dose oral amino acids for 12 weeks increased FFM in RA patients with rheumatoid cachexia [23].

During the last decade the use of food frequency questionnaires (FFQs) has become increasingly common to assess long-term dietary consumption. The accuracy of these has been proven in healthy individuals in terms of long-term dietary fat consumption, as this corresponds to fatty acid (FA) composition in adipose tissue [24-27].

The purpose of this study was to analyze if the type of diet over the previous year, determined by FFQ, was associated with body composition derangement and dyslipidemia in patients with RA. As a secondary goal, we also wanted to evaluate how rheumatoid cachexia relates to cardiovascular risk factors.

## Materials and methods

### Patients

A total of 80 consecutive outpatients with RA at the Rheumatology Department, Karolinska University Hospital Huddinge, Stockholm, Sweden were included in the study. Eligible patients were aged 18 to 80 years, had a diagnosis of RA [28] and had disease duration of ≥ 1 year. The exclusion criteria were: current malignancy, severe heart failure according to the New York Heart Association (NYHA) classification >3 [29], severe renal failure (glomerular filtration rate (GFR) <20 ml/min), chronic obstructive lung disease with emphysema, earlier gastric ulcer or intestinal surgery, known eating disorder or steroid injections within 2 weeks. The patient characteristics are shown in Table 1. Consequently, the patients had a fairly low disease activity according to mean Disease Activity Score (DAS28) [30] and a good functional ability as registered by Health Assessment Questionnaire (HAQ) [31]. Furthermore, 54% of the women and 68% of the men had hypertension, defined as a blood pressure above 140/90 or treatment with antihypertensive drugs [32].

**Table 1:** Patient characteristics

	**Women (n = 61)**	**Men (n = 19)**
Age, years	60.8 (57.3 to 64.4)	63.4 (59.8 to 66.9)
Disease duration, years^a^	6.0 (2.0 to 15.0)	5.0 (3.0 to 9.0)
Rheumatoid factor positive, N (%)	51 (84)	14 (74)
Erosive disease, N (%)	47 (77)	14 (74)
ESR, mm/h^a^	16.0 (9.0 to 29.0)	12.0 (9.0 to 15.0)
C-reactive protein, mg/l^a^	2.0 (1.0 to 8.0)	3.0 (1.0 to 9.0)
DAS28	3.3 (3.0 to 3.6)	2.6 (2.1 to 3.0)
HAQ score (0 to 3)	0.7 (0.5 to 0.8)	0.5 (0.2 to 0.7)
Patients on DMARDs, N (%)	59 (97)	19 (100)
Patients on glucocorticoids, N (%)	17 (28)	3 (16)
Glucocorticoids, dose, mg	4.3 (3.4 to 5.3)	4.2 (0.6 to 7.8)
Hypertension, N (%)	33 (54)	13 (68)
MetS, N (%)	12 (20)	12 (63)

Data is presented as mean (95% confidence intervals) for normally distributed variables and as median (interquartile range) for non-parametric variables.

^a^Median.

DAS28, 28-joint Disease Activity Score (where a value of >5.1 is regarded as high disease activity, <3.2 is regarded as low activity and <2.6 is regarded as remission); ESR, erythrocyte sedimentation rate; DMARD, disease-modifying antirheumatic drug (for example, methotrexate, sulfasalazine, hydroxychloroquine, cyclosporine, natriumaurotiomalat and azathioprine); HAQ, Health Assessment Questionnaire; MetS, metabolic syndrome.

The study was approved by the Ethics committee at Karolinska Institute, Stockholm, Sweden (reference number 2006/593-31/2), and was performed in accordance with the Helsinki declaration. Before entering the study the patients were given oral and written information, after which we received written informed consent from the patients.

### Dietary assessment

The self-administered, semiquantitative FFQ was designed to classify individuals according to levels of average daily intake of selected nutrients from food and dietary supplements [33]. Participants were asked to report their frequency of use of 88 food items over the past year. There were nine possible frequency categories in increasing order from never or almost never to three times per day. Furthermore, there were open-ended questions about the quantity of some food items eaten daily by most Swedes; for example, milk, bread, coffee, and cheese [33]. The nutrient calculations were carried out using nutrient composition values from the Swedish National Food Administration data [34]. The intake of nutrients was computed by multiplying the frequency of consumption of each food item by the nutrient content of the specified portions.

According to the Swedish National Food Administration the proportions (energy percentage (E%)) of carbohydrate, protein and fat should be 55 to 65 E%, 10 to 15 E% and 25 to 35 E%, respectively, for normally active individuals. For less active individuals the recommendations are 55 E%, 16 E% and 28 E%, respectively [35]. A Mediterranean diet was defined as a diet with high intake of fruits, vegetables, legumes, nuts, olive oil, fish, shellfish, a minimum of dairy products and lean meat [33].

### Biochemical measures

Venous blood samples were drawn between 07:30 and 10:00 after an overnight fast. The biochemical variables were determined by standard laboratory methods with commercial kits. They included: C-reactive protein (CRP), erythrocyte sedimentation rate (ESR), plasma glucose, total cholesterol, LDL, HDL and triglycerides. The serum lipid concentrations were considered pathological when total cholesterol was >5.0 mmol/l, LDL ≥ 3.0 mmol/l, triglycerides ≥ 1.7 mmol/l, for women HDL <1.3 mmol/l and for men HDL <1.1 mmol/l.

The samples had also been stored at -70 C before analyses. OxLDL was determined by use of a commercial kit (Mercodia, Uppsala, Sweden) as per the manufacturer's instructions. Anti-PC IgM was also determined by use of a commercial kit (Athera CVDefine, Stockholm, Sweden) as per the manufacturer's instructions.

Subcutaneous abdominal fat cells were aspirated with a needle attached to a vacuum tube and stored at -70 C until analyzed. The FA composition of subcutaneous adipose tissue was analyzed by gas liquid chromatography as described previously [36]. The amounts of FA were given as the relative percentage of the sum of the FA analyzed.

### Body composition and metabolic syndrome

BMI was calculated from weight/height^2 ^(kg/m^2^). In accordance with World Health Organization (WHO) standards, individuals with BMI values <18.5 kg/m^2 ^are considered underweight, between 18.5 to 24.9 as normal, 25 to 29.9 as overweight and values greater than 30 indicate obesity [37].

Waist circumference (WC) was measured to identify centrally obese individuals. Waist was measured to the nearest 0.5 cm midway between the iliac crest and the lower rib margin.

According to the International Diabetes Federation (IDF), a waist circumference value less than 80 cm in women and 94 cm in men indicates low risk, 80–87.9 cm in women and 94–101.9 cm in men indicates intermediate risk, and a value above 88 cm in women and 102 cm in men high risk of type 2 diabetes, coronary heart disease or hypertension [38].

Metabolic syndrome (MetS) was defined according to the IDF [38]; that is, presence of central obesity (waist perimeter ≥ 80 cm in females and ≥ 94 cm in males) plus two of the following four criteria: hypertriglyceridemia (triglycerides ≥ 1.7 mmol/1 or specific treatment), unfavorable HDL level (<1.3 mmol/1 in females and <1.1 mmol/1 in males and or specific treatment), hypertension (systolic blood pressure (BP) ≥ 130 mmHg or diastolic BP ≥ 85 mmHg or specific antihypertensive treatment) and fasting plasma glucose ≥ 5.6 mmol/1 or prior diagnosis of type 2 diabetes mellitus.

Body composition was measured with total body dual energy x ray absorptiometry (DXA) (GE-Lunar Prodigy, software enCore 2006, v. 10.20.105, Madison, Wisconsin, USA,). FFM and FM were expressed in absolute kg, and FM also as percentage of total mass. The reference value for FM% is 20% to 30% for women and 12% to 20% for men [39].

Fat free mass index (FFMI, kg/m^2^) and fat mass index (FMI, kg/m^2^) were also calculated. Age-matched and sex-matched data from a Swiss population of healthy adults (2,986 men and 2,649 women) were used to classify low FFM or excess FM [40]. The cutoff value for low FFM was defined as FFMI below the 10th percentile and obesity was defined as FMI above the 90th percentile, as defined by the reference population [40].

As there is no established criterion for rheumatoid cachexia we used both the definition by Engvall *et al.*, who categorized the patients as rheumatoid cachectic if FFMI was below the 10th percentile and FMI above the 25th percentile [41], and also the definition composed of FFMI below the 25th percentile and FMI above the 50th percentile.

### Statistical analysis

Data were presented as mean (confidence interval) or median (interquartile range) depending on whether the data were normally distributed or not. Differences between patient groups were assessed using the Student t test and Mann-Whitney U test, depending on the distribution of the analyzed variable.

To evaluate the association between intake of fatty acids in the diet and their relative content in adipose tissue Spearman correlations were used.

FAs in adipose tissue are described as the percentage of the total FAs analyzed. Dietary FAs are described as the percentage of total fat intake as well as grams per day.

*P *values < 0.05 were considered significant. The statistical analysis program Statistica 7 (Stat Soft Scandinavia AB, Uppsala, Sweden), was used for statistical analysis.

## Results

### Dietary intake and fatty acid profiles

The mean dietary intake of total energy, carbohydrate, protein and fat is shown in Table 2. Compared with the Swedish Food Recommendations for individuals with low physical activity [35], the RA patients had a low intake of total energy. As to the diet composition, the patients reported lower carbohydrate intake and higher fat intake than recommended. The fat intake consisted of more saturated and less unsaturated fat than recommended.

**Table 2:** Energy intake according to food frequency questionnaire (FFQ) in rheumatoid arthritis (RA) patients and Swedish recommendations for low active individuals

	**Women with RA (n = 61)**	**Recommendations for females**	**Men with RA (n = 19)**	**Recommendations for males**
Energy, kcal	1,668 (1,537 to 1,799)	2,200	2,402 (2,123 to 2,680)	2,700
Carbohydrate, g	195 (178 to 212)	293	268 (238 to 298)	369
Carbohydrate, E%	48	55	45	55
Protein, g	73.0 (66.7 to 79.3)	86	103 (88.4 to 118)	107
Protein, g/kg	1.13 (1.01 to 1.23)		1.18 (1.0 to 1.36)	
Protein, E%	18	16	17	16
Total fat, g	61.1 (55.8 to 66.4)	69	88.6 (75.1 to 102.1)	87
Total fat, E%	33	28	33	28
SFA, g	26.4 (23.7 to 29.0)	20	39.1 (31.9 to 46.2)	30
SFA, E%	14	10	15	10
MUFA, g	21.0 (19.2 to 22.8)	30	30.9 (26.5 to 35.2)	40
MUFA, E%	11	11	12	11
PUFA, g	8.9 (7.9 to 9.8)	15 to 30	11.8 (9.8 to 13.8)	15 to 30
PUFA, E%	4.8	5	4.4	5
Omega 3 FA, g	1.9 (1.8–2.1)	≥ 3.0	1.7 (1.6–1.8)	≥ 3.0
Omega 3 FA, E%	1.0	1.3	0.6	1.3
Omega 6 FA, g	6.9 (6.2 to 7.7)	10.8	11.0 (10.4 to 11.5)	10.8
Omega 6 FA, E%	3.7	3.6	4.1	3.6

E%, energy percentage; FA, fatty acid; MUFA, monounsaturated fatty acid; PUFA, polyunsaturated fatty acid; SFA, saturated fatty acid.

Low total energy intake was mainly reported by patients with moderate inflammatory activity. Thus, female patients with DAS28 >3.2 had a lower mean energy intake than those with DAS28 ≤ 3.2 (1,500 kcal/day vs. 1,800 kcal/day, *P *= 0.016). The proportions (E%) of carbohydrate, protein and fat were similar in these two groups. Saturated fatty acid (SFA) intake was significantly associated with DAS28 (r = 0.26, *P *= 0.017).

In all, 65 patients (81%) reported eating a normal western diet (46 of the women and all of the men), 3 patients (all women) were vegetarians and 12 (all women) followed a Mediterranean-like diet. The reported dietary intake of FAs correlated significantly with that in adipose tissue as to FA 14:0 (myristic acid), 18:2 n-6 (linoleic acid), 18:3 n-3 (α-linolenic acid), 20:4 n-6 (arachidonic acid), 20:5 n-3 (eicosapentaenoic acid), 22:5 n-3 (docosapentaenoic acid) and 22:6 n-3 (docosahexaenoic acid), but not with 16:0 (palmitic acid) and 18:1 n-9 (oleic acid) (Table 3). The FA in adipose tissue consisted predominantly of 18:1 n-9 (oleic acid) 51%, 16:0 (palmitic acid), 22% and 18:2 n-6 (linoleic acid) 11%.

**Table 3:** Spearman correlation coefficients between relative (% of total fat) fatty acid (FA) diet intake calculated from food frequency questionnaire (FFQ) and FA in adipose tissue

	**14:0 (FFQ)**	**16:0 (FFQ)**	**18:1 n9 (FFQ)**	**18:2 n6 (FFQ)**	**18:3 n3 (FFQ)**	**20:4 n6 (FFQ)**	**20:5 n3 (FFQ)**	**22:5 n3 (FFQ)**	**22:6 n3 (FFQ)**
14:0, AT	0.27**								
16:0, AT		-0.12							
18:1 n9, AT			0.21						
18:2 n6, AT				0.50*					
18:3 n3, AT					0.48*				
20:4 n6, AT						0.64*			
20:5 n3, AT							0.71*		
22:5 n3, AT								0.78*	
22:6 n3, AT									0.82*

*P < 0.001, **P < 0.05

AT = adipose tissue.

When analyzing the adipose FA content for the different self-reported diet groups, it was found that subjects compliant to Mediterranean-like or vegetarian diets had a more favorable FA profile; that is, with higher polyunsaturated fatty acids (PUFAs) than those reporting to eat a normal Western diet, Table 4.

**Table 4:** Fatty acids in adipose tissue and serum levels of lipids and anti-PC IgM in women with rheumatoid arthritis (RA), separated for diet

	Normal diet (n = 46)	Mediterranean-like diet (n = 12)	*P *value	Mediterranean-like and vegetarian diets (n = 15)	*P *value
Adipose tissue:					
SFA	29.2 (28.4 to 29.9)	29.1 (27.5 to 30.7)	0.60	28.8 (27.5 to 30.1)	0.90
14:0	3.3 (3.1 to 3.5)	3.4 (3.1 to 3.8)	0.60	3.5 (3.2 to 3.8)	0.43
16:0	21.7 (21.2 to 22.2)	21.7 (20.4 to 23.1)	0.92	21.4 (20.2 to 22.5)	0.55
MUFA	57.4 (56.7 to 58.1)	56.1 (54.6 to 57.6)	0.06	55.8 (54.4 to 57.2)	**0.016**
16:1 n7	6.5 (6.0 to 6.9)	6.0 (5.3 to 6.6)	0.30	5.8 (5.2 to 6.4)	0.13
18:1 n9	51.4 (50.9 to 52.0)	50.2 (48.9 to 51.2)	**0.05**	50.0 (49.0 to 51.0)	**0.019**
Omega 3	1.8 (1.7 to 1.9)	2.1 (1.7 to 2.6)	**0.039**	2.2 (1.8 to 2.6)	**0.009**
18:3 n3	1.1 (1.0 to 1.2)	1.1 (0.93 to 1.2)	0.72	1.1 (1.0 to 1.3)	0.22
20:5 n3	0.14 (0.12 to 0.16)	0.19 (0.12 to 0.25)*	0.08	0.15 (0.11 to 0.25)*	0.16
22:5 n3	0.33 (0.29 to 0.37)	0.40 (0.31 to 0.50)	0.06	0.39 (0.29 to 0.48)	0.11
22:6 n3	0.32 (0.28 to 0.36)	0.47 (0.25 to 0.59)*	0.07	0.47 (0.20 to 0.60)*	0.13
Omega 6	11.0 (10.6 to 11.3)	12.0 (10.7 to 13.2)	0.06	12.6 (11.2 to 14.1)	**0.004**
18:2 n6	10.1 (9.7 to 10.5)	11.2 (9.8 to 12.5)	0.06	11.9 (10.4 to 13.4)	**0.003**
20:4 n6	0.40 (0.36 to 0.44)	0.39 (0.31 to 0.46)	0.81	0.32 (0.26 to 0.45)*	0.35
Serum lipids:					
Cholesterol, mmol/l	5.3 (5.0 to 5.6)	5.1 (4.7 to 5.6)	0.60	5.1 (4.8 to 5.5)	0.57
HDL, mmol/l	1.9 (1.7 to 2.0)	1.9 (1.4 to 2.4)	0.97	1.9 (1.5 to 2.3)	0.86
LDL, mmol/l	2.9 (2.7 to 3.2)	2.8 (2.3 to 3.2)	0.58	2.8 (2.3 to 3.2)	0.52
OxLDL, U/l	62.1 (55.7 to 68.4)	59.4 (50.0 to 68.9)	0.67	58.8 (51.2 to 66.3)	0.57
Triacylglycerol, mmol/l	0.98 (0.88 to 1.1)	1.0 (0.82 to 1.2)	0.63	1.0 (0.81 to 1.1)	0.95
anti-PC IgM, U/ml_a_	43.5 (30.3 to 65.5)	198.2 (171.6 to 209.2)	**<0.001**	188.2 (63.3 to 208.1)	**<0.001**

Values are means (95% confidence interval) and ^a^medians (interquartile range). The fatty acids in adipose tissue are expressed as a percentage of total fat. Significant results are in bold.

Anti-PC IgM, antibodies against phosphorylcholine IgM; FA, fatty acid; HDL< high-density lipoprotein; LDL, low-density lipoprotein; MUFA, monounsaturated fatty acid; oxLDL, oxidized low-density lipoprotein; PUFA, polyunsaturated fatty acid; SFA, saturated fatty acid.

### Serum lipids and anti-PC IgM

Lipid mean values for women with RA with normal Western diet and with the Mediterranean-like/vegetarian diets are presented in Table 4. There were no significant differences between the diet groups. A total of 53% of the women and 32% of the men had cholesterol >5.0 mmol/l. Low HDL levels were found in 3% of the women and 5% in the men. High LDL levels were present in 51% of the women and in 32% of the men. Corresponding figures for triglycerides were 1% and 37% respectively. Concerning oxLDL, no reference values exist.

However, anti-PC IgM levels differed significantly between patients in the different self-reported diet groups. Women with Mediterranean-like diet had significantly higher median level compared with those with self-reported normal Western diet (p < 0.001), Table 4.

### Anthropometrical assessments and body composition measurement

As shown in Table 5 the change in body weight from start of RA disease to the present investigation was fairly small for both sexes. BMI indicated that both women and men were overweight. Only one of the patients had BMI <18.5. A total of 55% of the patients had a BMI >25. Central obesity was found in 57% of the women (WC >80 cm) and in 89% of the men (WC >94 cm). In all, 20% of the women and 63% of the men could be diagnosed to have MetS according to IDF guidelines [38].

**Table 5:** Anthropometry and body composition measurement in rheumatoid arthritis (RA) patients

	Women (n = 61)	Men (n = 19)
Weight change since disease onset, kg	2.3 (-0.8 to 5.9)	4.4 (-2.2 to 8.0)
BMI, kg/m^2^	25.0 (23.6 to 26.3)	27.0 (25.7 to 29.1)
Waist circumference, cm	85 (81 to 89)	105 (100 to 110)
Central obesity (WC >80 cm in women, >94 cm in men), N (%)	35 (57)	17 (89)
FM, total, kg	25.8 (22.8 to 28.7)	27.9 (23.4 to 32.5)
FM, total, %	37.8 (35.7 to 39.9)	31.7 (28.1 to 35.2)
FMI, kg/m^2^	8.8 (6.5 to 11.4)^a^	8.6 (7.2 to 9.9)
FFM, total, kg	42.0 (40.6 to 43.5)	61.2 (58.6 to 63.8)
FFMI, kg/m^2^	15.5 (15.1 to 15.9)	18.9 (18.2 to 19.6)

Data is presented as mean (95% confidence interval) for normally distributed variables and as median (interquartile range) for non-parametric variables.

^a^Median

BMI, body mass index; FFM, fat free mass; FFMI, fat free mass index; FM, fat mass; FMI, fat mass index; WC, waist circumference.

Body composition data are shown in Table 5. In all, 31% of the women and 53% of the men had a FMI above the 90th percentile of the reference population, implying that they could be classified as obese. Reduced FFMI, that is, below the 10th percentile of a reference population, was registered in 26% and 21% of the women and men, respectively. Furthermore, 18% of the women and 26% of the men were assessed to have rheumatoid cachexia when using the definition of FFMI below the 25th percentile and FMI above the 50th percentile. By the definition of FFMI below the 10th percentile and FMI above the 25th percentile, 18% and 21% of the women and men, respectively, were assessed to have rheumatoid cachexia. FFM correlated with disease duration (r = -0.30, p = 0.008), in contrast to FM.

### Relationship between body composition and serum lipids as well as anti-PC antibodies and inflammatory markers

Patients with low FFMI, below the 10th or the 25th percentile of healthy adults, had serum lipid and anti-PC levels similar to the rest of the patients [Additional data file 1]. Patients with FMI above the 50th percentile had lower HDL and higher triglycerides than the other patients. When combining low FFMI and high FMI according to the two proposed definitions of rheumatoid cachexia, it was evident that the patients with FFMI <25th percentile in combination with FMI >50th percentile had higher LDL, cholesterol and trendwise oxLDL, as well as significantly lower values of anti-PC IgM (p = 0.040), than those without this derangement in their body composition.

With regard to DAS28 and CRP, there were no significant differences between any of the body composition groups (as defined in [Additional data file 1]) and those not belonging to the respective group. However, when separating the patients according to the DAS28 level it was found that patients with DAS28 ≥ 3.2 had significantly higher FM% (38.0 vs. 35.3, p = 0.032), lower FFM (43.5 vs. 48.5 kg, p = 0.018) and lower FFM/FM ratio (1.6 vs. 2.1, p = 0.031) than those with DAS28 ≤ 3.2.

### Relationship between body composition and diet groups as well as presence of hypertension and MetS

There were no significant differences in intake of energy or fat (total SFA or total PUFA, also separated by total omega 3 and omega 6) between those who were considered to have rheumatoid cachexia or not, irrespective of definition. Neither did the frequency of patients with the self-reported Mediterranean and vegetarian diets differ between these groups (data not shown).

Patients with FMI above the 50th percentile and patients with rheumatoid cachexia, defined as FFMI <25th percentile in combination with FMI >50th percentile, had the highest frequencies of hypertension and MetS [Additional data file 1]. Furthermore, we found that patients with hypertension had significantly lower values of anti-PC IgM, median (interquartile range (IQR)) of 39.9 (30.1 to 73.8), than those with normal blood pressure, median (IQR) of 54.5 (41.6 to 160.7) (p = 0.046). Treatment with glucocorticoids and mean dose given did not differ between those who were cachectic and those not (data not shown).

## Discussion

This study shows that ambulatory patients with RA of low disease activity were reported to have a diet intake high in saturated fat. They were in general centrally obese and a fifth had rheumatoid cachexia. A large proportion of the patients had hypertension and MetS, especially those with high FM and rheumatoid cachexia, conditions associated with high levels of oxLDL and low levels of anti-PC. Patients compliant with Mediterranean and vegetarian diets did not differ in body composition from the rest of the patients, but had a higher content of PUFA in adipose tissue and significantly higher serum levels of the atheroprotective anti-PC.

The patients had, according to the FFQ, a lower than recommended caloric intake. It is unclear if this was a real difference or a consequence of underreporting. However, underreporting seems most probable as earlier comparisons between FFQ and weighed diet records concerning fat intake showed lower total intake registered by FFQ than the weighed diet, whereas the proportions of fat components were similar [27].

With regard to the caloric proportions of the diet, our patients had a higher intake of saturated fat and lower intake of unsaturated fat compared to the Food Recommendations in Sweden [35]. A high fat intake has also been noted in RA patients in the USA, of which 40% were obese [21].

The reliability of the long-term fatty acid intake according to the FFQ was verified by the significant correlations found between the PUFAs ingested and those in adipose tissue. PUFAs in adipose tissue are largely exogenous and hence valid markers to assess dietary intake [42,43]. By contrast, SFAs and monounsaturated fatty acids (MUFAs) are not considered to be good biomarkers of intake because they are also endogenously synthesized [42,44], which was evident in the RA patients with no significant correlations concerning 16:0 and 18:1 n-9. Furthermore, the noted significant correlation between 14:0 FA in diet and adipose tissue verifies a report by Wolk *et al. *that this FA is also a valid biomarker for FA intake [45].

When comparing the proportions of FAs in the adipose tissue in our RA patients with healthy subjects from different countries (Sweden, Denmark, USA, Spain and Costa Rica), our RA patients had higher proportions of SFA and lower proportions of PUFA than healthy subjects from the above-mentioned countries [25,42,43,46,47]. However, the SFA and PUFA content of adipose tissue was similar to that described in RA patients from the south of Sweden [48].

The patients reported in general a high intake of saturated fat. This high intake was also reflected in the FA composition of the adipose tissue. However, we did not find that the FA intake differed between cachectic and non-cachectic patients. Adherence to a Mediterranean diet did not differ either between cachectic and non-cachectic patients. Thus, diet as analyzed here does not seem to cause the derangement of body composition leading to cachexia. This finding is in accordance with the known difficulties of treating cachexia with diet. However, recently supplements with amino acids have been shown to increase muscle mass [23]. By contrast, the high intake of dietary fat may have contributed to the dyslipidemia displayed by the patients.

With regard to dietary therapies, 33% to 75% of RA patients believe that food plays an important role in their disease, and 20% to 50% have tried dietary manipulation in an attempt to relieve symptoms [49,50]. The present finding that dietary intake of SFAs was significantly associated with disease activity focuses on the immunosuppressive effect of unsaturated FAs. In the scientific community there is, however, no consensus on dietary recommendations to patients with RA. Evidence may emerge that, for patients at an advanced disease stage, a high-protein diet may be recommended and, for patients with early RA, restriction of excessive energy intake may be beneficial to prevent obesity [51]. A scientific basis for a role of dietary therapy in RA has grown in the last few years, although there is still no agreement on the nature of the optimum diet [52].

Several of the RA patients had dyslipidemia, hypertension and MetS, most frequently patients with high FM and rheumatoid cachexia. Of the lipids, oxLDL might be especially important as it has proinflammatory and immune stimulatory properties [53,54]. It has previously been detected in synovia from RA patients [55], and is suggested to be of importance in the pathogenesis of CVD [8]. Even though there are no existing reference values for oxLDL, the present patients had higher levels than earlier reported for healthy people, with mean (SD) values of 42.2 U/l (± 14.7) and mean (95% confidence interval (CI)) 48 U/l (35 to 68) [7,56]. Additionally, when compared with RA patients from Korea who had a mean (SD) value of 53.0 U/l (± 20.9) [7], our patients had higher levels, a difference that might be secondary to different diet habits.

One novel finding herein is that anti-PC levels decreased in patients with rheumatoid cachexia. Low anti-PC levels could predispose patients to cardiovascular disease [10,11] and might thus be one explanation for the increased morbidity in rheumatoid cachexia. The cause of this association is not known.

Also, the very high levels of anti-PC in patients on a Mediterranean diet is of great interest, as patients on this diet are reported to have a lower frequency of CVD [57,58]. In RA anti-PC have not been studied in relation to CVD, but we have recently shown that the levels of anti-PC in serum increased in RA patients when changing from a normal Western diet to a gluten-free vegan diet [12]. The present finding adds strength to our previous report, indicating that diet may influence atheroprotective anti-PC in RA.

A high frequency of MetS has previously been reported in RA patients [59-61]. In these patients an association between MetS and disease activity, as well as coronary calcification, has been shown [60,61], suggesting that the increased prevalence of coronary heart disease in RA patients may, at least in part, be attributed to inflammation and change of fat metabolism.

In the present RA population every fourth to fifth patient had rheumatoid cachexia, irrespectively of which of the two definitions for cachexia were used. There is no standard definition of this condition as reviewed by Summers *et al*. [62], which is why the frequency of rheumatoid cachexia varies. The cachexia and muscle wasting found here could not reflect the changes in body composition that occur with age [63], as the reference population was matched for age and sex [40]. Age-related muscle wasting is a separate condition named sarcopenia. Cachexia in chronic inflammatory diseases is suggested to be associated with increased morbidity [19]. Mostly, the morbidity has been attributing the loss of body lean mass [13], whereas the coincidental increase of fat mass has not been considered in this context. In the present study it was obvious that FM above the 50th percentile was, together with FFM below the 25th percentile, associated with dyslipidemia as were increased levels of total cholesterol, LDL and oxLDL and also low levels of anti-PC. These patients are therefore suggested to have an increased risk of CVD and premature mortality [10,11,64]. In fact, they also had high frequencies of hypertension and MetS.

Low FFM alone, irrespective of FM, was not associated with these risk factors for CVD. By contrast, only one of the patients was severely emaciated with extremely low BMI known to be associated with cardiovascular mortality in RA [65].

A large proportion of the patients were centrally obese, which is in line with earlier reports [16]. This fact might contribute to the increased presence of risk factors for CVD in the patients with rheumatoid cachexia.

In the present cohort of patients we did not find that DAS28 or CRP differed between those with cachexia and the rest of the patients, probably related to the fact that most patients had low inflammatory activity. However, when separating the patients into those with low DAS28 and those with DAS28 corresponding to moderate activity it was found that the latter had lower FFM and higher FM than the former. Thus, the inflammation *per se *might have contributed to the derangement in body composition found here and also described previously [18,41,66]. A further explanation could be that patients with DAS28 of moderate activity reported lower energy intake than those with low activity.

## Conclusion

In our cohort of RA patients with low disease activity and BMI in the upper reference range every fourth to fifth patient had rheumatoid cachexia, a condition that was not associated with the observed increased intake of fatty acids. Rheumatoid cachexia defined as FFMI below the 25th percentile and FMI above the 50th percentile was associated with risk factors for CVD, including raised cholesterol and LDL and also decreased levels of the atheroprotective marker anti-PC as well as a high frequency of hypertension. We thus propose this definition of rheumatoid cachexia to be used, as it verifies that it is associated with increased morbidity. However, patients on a Mediterranean-like diet had high anti-PC levels in spite of a similar frequency of cachexia, which may provide some protection against CVD events.

## Abbreviations

Anti-PC: antibodies against phosphorylcholine; AT: adipose tissue; BMI: body mass index; BP: blood pressure; CRP: C-reactive protein; CVD: cardiovascular disease; DAS28: 28-joint Disease Activity Score; DXA: dual energy x ray absorptiometry; ESR: erythrocyte sedimentation rate; FA: fatty acid; FFM: fat free mass; FFMI: fat free mass index; FFQ: food frequency questionnaire; FM: fat mass; FMI: fat mass index; HAQ: Health Assessment Questionnaire; HDL: high-density lipoprotein; IDF: International Diabetes Federation; LDL: low-density lipoprotein; MetS: metabolic syndrome; MUFA: monounsaturated fatty acids; oxLDL: oxidized low-density lipoprotein; PC: phosphorylcholine; PUFA: polyunsaturated fatty acid; RA: rheumatoid arthritis; SFA: saturated fatty acids; WC: waist circumference.

## Competing interests

The authors declare that they have no competing interests.

## Authors' contributions

ACE was responsible for the design of the study, for all measurements, for analyzing the data and was responsible for writing the manuscript. NH had responsibility for analyzing the FFQ. JF was responsible for assays of lipids and anti-PC antibodies. TC had an active role in the design of the study, in analyzing adipose tissue and an advisory role. IH took part in the design of the study and has been senior advisor in all parts of the research and manuscript preparation. All authors read and approved the final manuscript.

## Supplementary Material

Additional file 1 A table in Word format listing blood lipids, inflammatory marker, hypertension and metabolic syndrome in relation to different categories of body composition. Click here for file
