# Supplementary material for: Rheumatoid cachexia is associated with dyslipidemia and low levels of atheroprotective natural antibodies against phosphorylcholine but not with dietary fat in patients with rheumatoid arthritis: a cross-sectional study
Source: Arthritis Res Ther. 2009 Mar 10;11(2):R37. doi: 10.1186/ar2643 (PMC2688183; doi:10.1186/ar2643)
Supplement: Additional file 1 — A table in Word format listing blood lipids, inflammatory marker, hypertension and metabolic syndrome in relation to different categories of body composition. [file ar2643-S1.doc]

**Additional file.** Blood lipids, inflammatory marker, hypertension and metabolic syndrome in relation to different categories of body composition.

|  | **FFMI <P10**  **N=20** | **p-value** | **FFMI <P10 +FMI>P25**  **N=15** | **p-value** | **FFMI <P25**  **N=32** | **p-value** | **FMI >P50**  **N=53** | **p-value** | **FFMI <P25 + FMI >P50**  **N=16** | **p-value** |
| --- | --- | --- | --- | --- | --- | --- | --- | --- | --- | --- |
| **Blood lipids** |  |  |  |  |  |  |  |  |  |  |
| Cholesterol, mmol/l | 5.4 (5.0-5.8) | 0.20 | 5.4 (4.9-5.8) | 0.40 | 5.5 (5.2-5.9) | 0.08 | 5.1 (4.8-5.4) | 0.27 | 5.6 (5.1-6.2) | **0.033** |
| HDL, mmol/l | 1.9 (1.7-2.2) | 0.20 | 1.9 (1.6-2.2) | 0.19 | 1.9 (1.8-2.1) | 0.07 | 1.6 (1.5-1.8) | **0.003** | 1.8 (1.5-2.1) | 0.65 |
| LDL, mmol/l | 3.0 (2.6-3.4) | 0.52 | 2.9 (2.4-3.3) | 0.94 | 3.1 (2.8-3.4) | 0.18 | 2.9 (2.6-3.1) | 0.91 | 3.3 (2.8-3.7) | **0.029** |
| OxLDL, U/l | 67.2 (57.5-76.8) | 0.16 | 67.2 (55.1-79.3) | 0.24 | 65.9 (58.4-73.3) | 0.30 | 61.8 (56.2-67.4) | 0.98 | 70.1 (57.5-82.7) | **0.056** |
| Triacylglycerol, mmol/l | 1.1 (0.89-1.4) | 0.92 | 1.2 (0.88-1.5) | 0.79 | 1.0 (0.85-1.2) | 0.12 | 1.3 (1.1-1.5) | **0.017** | 1.1 (0.79-1.4) | 0.58 |
| **anti-PC IgM*, U/ml** | 43.7 (25.3-75.5) | 0.10 | 39.8 (19.8-73.9) | 0.10 | 41.6 (31.0-66.8) | 0.15 | 43.2 (55.8-103.6) | 0.23 | 38.5 (24.3-63.7) | **0.040** |
| Inflammation |  |  |  |  |  |  |  |  |  |  |
| DAS28 | 3.0 (2.5-3.5) | 0.61 | 2.6 (2.1-3.1) | 0.08 | 2.9 (2.5-3.4) | 0.27 | 3.1 (2.8-3.4) | 0.78 | 3.0 (2.4-3.6) | 0.68 |
| CRP*, mg/l | 3.5 (1.0-8.5) | 0.60 | 2.0 (1.0-6.0) | 0.78 | 2.0 (1.0-5.0) | 0.52 | 3.0 (1.0-9.0) | 0.09 | 2.5 (1.0-4.5) | 0.75 |
|  |  |  |  |  |  |  |  |  |  |  |
| **Hypertension N, (%)** | 11 (55) |  | 9 (60) |  | 15 (46) |  | 35 (66) |  | 11 (69) |  |
| **MetS, N, (%)** | 3 (15) |  | 3 (20) |  | 4 (13) |  | 23 (43) |  | 4 (25) |  |

Data are presented as mean (CI) for normally distributed variables and as median (inter-quartile range) for non-parametric variables,

*= median, N=numbers, %= percentage.

P values relate to the difference between the actual body composition category and patients without that derangement. FFMI<P10= fat free mass index below the 10^th^ percentile, FFMI<P25= fat free mass index below the 25^th^ percentile, FMI>P25= fat mass index above the 25^th^ percentile, FMI>P50= fat mass index above the 50^th^ percentile, MetS= metabolic syndrome.
